# Combining exome/genome sequencing with data repository analysis reveals novel gene–disease associations for a wide range of genetic disorders

**DOI:** 10.1038/s41436-021-01159-0

**Published:** 2021-04-19

**Authors:** Aida M. Bertoli-Avella, Krishna K. Kandaswamy, Suliman Khan, Natalia Ordonez-Herrera, Kornelia Tripolszki, Christian Beetz, Maria Eugenia Rocha, Alize Urzi, Ronja Hotakainen, Anika Leubauer, Ruslan Al-Ali, Vasiliki Karageorgou, Oana Moldovan, Patrícia Dias, Amal Alhashem, Brahim Tabarki, Mohammed A. Albalwi, Abdulrahman Faiz Alswaid, Zuhair N. Al-Hassnan, Malak Ali Alghamdi, Zahra Hadipour, Fatemeh Hadipour, Nadia Al Hashmi, Lihadh Al-Gazali, Huma Cheema, Maha S. Zaki, Irina Hüning, Ahmed Alfares, Wafaa Eyaid, Fuad Al Mutairi, Majid Alfadhel, Fowzan S. Alkuraya, Nouriya Abbas Al-Sannaa, Aisha M. AlShamsi, Najim Ameziane, Arndt Rolfs, Peter Bauer

**Affiliations:** 1 CENTOGENE GmbH, Rostock, Germany; 2 Serviço de Genética Médica. Hospital de Santa Maria. Centro Hospitalar Universitário de Lisboa Norte, Lisboa, Portugal; 3 Division of Pediatric Genetics, Department of Pediatrics, Prince Sultan Military Medical City, Riyadh, Saudi Arabia; 4 Pathology and Laboratory Medicine, King Abdulaziz Medical City, Riyadh, Saudi Arabia; 5 King Abdullah International Medical Research Center (KAIMRC), King Saud bin Abdulaziz University for Health Sciences, MNGHA, Riyadh, Saudi Arabia; 6 College of Medicine, King Saud bin Abdulaziz University for Health Sciences. King Abdulaziz Medical City, Riyadh, Saudi Arabia; 7 Division of Genetics, Department of Pediatrics, King Abdullah Specialized Children Hospital, King Abdulaziz Medical City, MNGHA, Riyadh, Saudi Arabia; 8 Department of Medical Genetics, King Faisal Specialist Hospital & Research Center, College of Medicine, Alfaisal University, Riyadh, Saudi Arabia; 9 Medical Genetic Division, Pediatric Department, College of Medicine, King Saud University, Riyadh, Saudi Arabia; 10 Medical Genetics Department, Atieh Research Center & Hospital, Tehran, Iran; 11 National Genetic Center, Royal Hospital Muscat. Sultanate of Oman, Muscat, Oman; 12 Department of Pediatrics, Tawan Hospital, Al-Ain, United Arab Emirates; 13 Pediatric Department of Gastroenterology, Children’s Hospital of Lahore Hospital, Lahore, Pakistan; 14 Clinical Genetics Department, Human Genetics and Genome Research Division, National Research Centre, Cairo, Egypt; 15 Institute of Human Genetics, University Hospital Schleswig-Holstein, Lübeck, Germany; 16 Department of Pediatrics, College of Medicine, Qassim University, Riyadh, Saudi Arabia; 17 Department of Genetics, King Faisal Specialist Hospital and Research Center, Riyadh, Saudi Arabia; 18 John Hopkins Aramco Health Care, Pediatric Services, Dhahran, Saudi Arabia; 19 University of Rostock, Rostock, Germany

## Abstract

**Purpose:**

Within this study, we aimed to discover novel gene–disease associations in patients with no genetic diagnosis after exome/genome sequencing (ES/GS).

**Methods:**

We followed two approaches: (1) a patient-centered approach, which after routine diagnostic analysis systematically interrogates variants in genes not yet associated to human diseases; and (2) a gene variant centered approach. For the latter, we focused on de novo variants in patients that presented with neurodevelopmental delay (NDD) and/or intellectual disability (ID), which are the most common reasons for genetic testing referrals. Gene–disease association was assessed using our data repository that combines ES/GS data and Human Phenotype Ontology terms from over 33,000 patients.

**Results:**

We propose six novel gene–disease associations based on 38 patients with variants in the *BLOC1S1*, *IPO8*, *MMP15*, *PLK1*, *RAP1GDS1*, and *ZNF699* genes. Furthermore, our results support causality of 31 additional candidate genes that had little published evidence and no registered OMIM phenotype (56 patients). The phenotypes included syndromic/nonsyndromic NDD/ID, oral–facial–digital syndrome, cardiomyopathies, malformation syndrome, short stature, skeletal dysplasia, and ciliary dyskinesia.

**Conclusion:**

Our results demonstrate the value of data repositories which combine clinical and genetic data for discovering and confirming gene–disease associations. Genetic laboratories should be encouraged to pursue such analyses for the benefit of undiagnosed patients and their families.

## INTRODUCTION

More than half of patients with genetic diseases remain undiagnosed, even after conducting genome-wide diagnostic approaches, such as exome and genome sequencing.^1,2^ Despite recent technological advances, the challenge of variant interpretation remains, in part due to the missing gene–phenotype link.^3^

The methods applied for the identification of causal gene defects for monogenic diseases have changed drastically in the last 10 years. Genome-wide scans using polymorphic microsatellite markers or single-nucleotide variants followed by linkage analysis were the predominant genetic mapping approach used in the past.^4^ This changed dramatically after the implementation and routine application of exome/genome sequencing in genetic research. Currently, most family-based approaches for disease gene identification rely on the analysis of exome or genome data. Study designs vary from including single unrelated individuals with a similar phenotype to typical family-based studies with the inclusion of several affected and unaffected relatives to focus on regions of homozygosity or using the de novo approach.^5^ Furthermore, phenocentric (focused on specific patients and phenotypes) and genocentric (focused on database analyses and algorithms) approaches have been described.^6,7^ Identification of candidate genes/variants associated with disease is usually followed by replication in other unrelated, similarly affected patients and/or functional studies to validate variants’ pathogenicity.^5^

The unambiguous assignment of disease causality is often difficult to achieve, and, in many cases, the initially collected evidence is insufficient to prove causality. The rarity, severity, and clinical heterogeneity of many genetic disorders complicates the process of finding additional patients. Furthermore, the lack of knowledge on the gene/protein function challenges the final assignment of gene causality. Thus, the gene candidacy remains inconclusive and is considered as a research gene.

Within this study, we analyzed exome/genome data together with the respective clinical phenotypes of the patients using Human Phenotype Ontology (HPO) to identify novel gene–disease associations and to validate previously reported candidate genes. We present six novel gene–disease associations and the confirmation of 31 additional candidate genes. The outcome has substantial implications for the diagnosis and counseling of the patients and their families.

## MATERIALS AND METHODS

### Patients

Written informed consent included several sections: consent for genetic testing related to the disease(s) of the patient, and consent for research (related to the main concern, but implicating genes not yet associated to human diseases). Additionally, the consent declaration included information regarding storage of the data and further processing for research purposes. Written informed consent was given by patients, parents, or referring physicians. Consent for scientific publication of patient photographs was obtained as well. Data regarding country of origin, family history, consanguinity, clinical phenotype, and previous genetic testing were extracted from our database.

### Exome and genome sequencing (ES/GS)

DNA was extracted from EDTA blood or from dried blood spots on filter cards (CentoCard®) using standard, spin column-based methods.

ES was performed as previously described.^2^ In short, the Nextera Rapid Capture Exome Kit (Illumina, San Diego, CA), the SureSelect Human All Exon kit (Agilent, Santa Clara, CA) or the Twist Human Core Exome was used for enrichment, and a Nextseq500, HiSeq4000, or Novoseq 6000 (Illumina) instrument was used for the actual sequencing, with the average coverage targeted to at least 100× or at least 98% of the target DNA covered 20×. When carrying out GS, genomic DNA was fragmented by sonication, and Illumina adapters were ligated to generated fragments for subsequent sequencing on the HiSeqX platform (Illumina) to yield an average coverage depth of at least 30×. Data analysis, including base calling, de-multiplexing, alignment to the hg19 human reference genome (Genome Reference Consortium GRCh37), and variant calling, was performed using the HiSeq Analysis Software v2.0 pipelines (Illumina, Inc., San Diego, CA), as previously described^8^ (Supplementary information).

Variants with suboptimal quality were confirmed via Sanger sequencing according to our established criteria^9^ or quantitative polymerase chain reaction (qPCR), multiplex ligation-dependent probe amplification (MLPA), or chromosomal microarray (CMA) for copy-number variations (CNVs). An extended Methods section can be found in the Supplementary Information.

### Variant evaluation and classification

The clinical information was translated into HPO terms, registered in our data repository, and applied for each individual analysis during variant filtration and prioritization as previously described.^2,10^ Variant nomenclature followed standard Human Genome Variation Society (HGVS) recommendations.^11^ Variants in established diagnostic genes were classified according to the published guidelines of the American College of Medical Genetics and Genomics (ACMG) and Association for Molecular Pathology (AMP) as pathogenic (P), likely pathogenic (LP), and variant of unknown significance (VUS).^12^

**Fig. 1:**
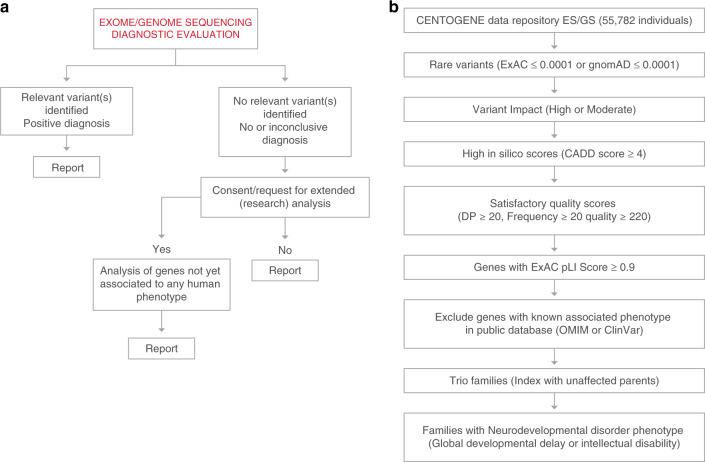
Summary of the applied strategies for identification of novel gene–disease associations. **a** Patient-centered approach to systematically interrogate variants in genes not yet associated to human diseases in patients with no genetic diagnosis after exome/genome sequencing (ES/GS). **b** Gene variant centered approach to analyze de novo variants in cases with ES/GS performed and no genetic diagnosis. DP depth of reads, pLI probability of loss-of-function intolerance.

For patients with no relevant variant(s) identified during the diagnostic process, a second analysis was conducted with the aim of identifying variants in genes not yet associated to any human phenotype. The results were reported to the referring physician as research findings in a dedicated section of the genetic report. The workflow is summarized in Fig. 1a.

### Analysis of own data repository

Patients’ reports containing research findings were retrieved from the database (2016–2019). Reported variants were reassessed taking into consideration current knowledge on the gene function and compatibility with the patient phenotype (e.g., based on animal models). Only cases with negative or inconclusive (VUS) diagnostic findings were included (Fig. 1a). As a second step, the data repository was queried for other rare variants in the respective candidate gene and the overlapping clinical features of the individuals.

Our data repository (CentoMD®)^13^ contains ES/GS data from 55,782 individuals (50,023 ES/5,759 GS), of whom 33,280 individuals (29,842 ES/3,438 GS) have clinical descriptions that include at least one HPO term. Neurodevelopmental delay (NDD) or intellectual disability (ID) are among the most frequent reasons for genetic consultation and testing. Thus, a second, gene-centered approach was applied to identify de novo variants in patients with NDD/ID. Variants that are rare in external databases (ExAc ≤0.0001 or gnomAD ≤0.0001) and have a high or moderate predicted impact on protein structure or function (missense, affecting splicing sites, nonsense, frameshift, indels) and high CADD raw score (above 4) were prioritized. Only variants with satisfactory quality scores were considered (read depth ≥20, frequency ≥20 and quality score ≥220).^9^ In addition, variants mapping to 3,230 genes with high probability of loss-of-function intolerance (pLI) scores ExAC calculations of pLI ≥0.90) were prioritized. Genes with an associated clinical phenotype in OMIM or ClinVar were excluded from this analysis. Finally, only index cases with parental samples available who had no established genetic diagnosis during former ES/GS evaluations were included. Figure 1b summarizes the filtering strategy.

## RESULTS

We applied two different strategies to identify novel gene–disease associations. For the first approach (patient-centered), we extended the ES/GS evaluation to genes with no known disease association, according to the OMIM database. A summary of the implemented workflow is shown in Fig. 1a. In using this strategy, we identified 191 candidate genes in patients with a wide range of clinical phenotypes. Furthermore, we used a second approach (gene-centered) oriented toward identifying unreported, de novo variants in patients with NDD/ID. We focused on NND/ID as these are among the main reasons for genetic testing referrals. The main parameters applied are summarized in Fig. 1b. We identified 287 candidate genes using this approach.

Then, we reviewed the evidence supporting variant/gene pathogenicity and individual patient data. We took into consideration the OMIM database, PubMed, Uniprot, and the Human Protein Atlas. With this evaluation, we detected genes that were already recognized by us as candidates and for which independent publications were ongoing, for example *ADAMTS19*^14^ and *EMC10*.^15^ Additional genes had recently been published as causal for genetic disorders, such as *FBXW11*,^16^ *GRIA2*,^17^ *PPP1R21*,^18^ and *TAOK1*, which was recently published by us.^19^ Other genes such as *TANC2*^20^ and *NEK10*^21^ were published as causative in the months following our initial analysis and during the preparation of this paper. These examples can be considered as a proof of principle, confirming the effectiveness of the applied approaches.

For the identification of novel gene–disease associations, we focused on genes with more than one hit and no previous association to a human disease. We selected genes with variants in at least two unrelated cases and published genetic or functional evidence indicating a role in disease, or with at least three unrelated patients if there was limited available evidence on gene function. This analysis enabled the identification of novel gene–disease associations based on 38 patients with variants in six genes: *BLOC1S1*, *IPO8*, *MMP15*, *PLK1*, *RAP1GDS1*, and *ZNF699* (Table 1).

**Table 1:** Variants from six novel gene–disease associations.

Patient	Gene	cDNA	Protein	Zygosity	Consanguinity, family history	Phenotype	Evidence	PMID	Others
**1**	*BLOC1S1*	NM_001487.3:c.284C>A	p.Ala95Asp	Hom	Yes, yes	Abnormal myelination, generalized-onset seizure, intellectual disability, leukodystrophy, optic atrophy, seizures, severe global developmental delay, spasticity	BLOC1S1 is a component of the ubiquitously expressed BLOC1 multisubunit protein complex. Loss-of-function variants in two of the genes coding for proteins that form the complex, DTNBP1 and BLOC1S3, cause Hermansky–Pudlak syndrome. Mutant flies lacking the conserved Blos1 subunit have eye pigmentation defects due to abnormal pigment granules (lysosome-related organelles), and abnormal glutamatergic transmission and behavior	15102850, OMIM 614076 and 614077	Unaffected sister is heterozygous
**2**	*BLOC1S1*	NM_001487.3:c.284C>A	p.Ala95Asp	Hom	Yes, yes (sibling above)	Abnormal myelination, generalized-onset seizure, intellectual disability, leukodystrophy, optic atrophy, seizures, severe global developmental delay, spasticity	As above	As above	As above
**3**	*BLOC1S1*	NM_001487.3:c.185T>C	p.Leu62Pro	Hom	Yes, no	Delayed myelination, cerebral hypomyelination, severe intellectual disability, severe global developmental delay, epilepsy, spastic tetraparesis, dystonia, severe visual impairment, optic atrophy	As above	As above	–
**4**	*BLOC1S1*	NM_001487.3:c.218 + 3A>G (NC_000012.11:g.56110792A>G)	p.?	Hom	Yes, yes	Arachnodactyly, congenital onset, developmental regression, global developmental delay, intellectual disability, leukodystrophy, leukoencephalopathy, muscular hypotonia, talipes equinovarus	As above	As above	–
**5**	*IPO8*	NM_006390.3:c.700C>T	p.Arg234*	Hom	Yes, no	Hydronephrosis, high palate, cupped ear, prominent nasal bridge, long palpebral fissure, ectropion, pectus carinatum, atopic dermatitis, muscular hypotonia, global developmental delay, joint hypermobility, failure to thrive, umbilical hernia, alopecia, ventricular septal defect, patent ductus arteriosus, double outlet right ventricle, talipes equinovarus, abnormal facial shape, chronic diarrhea, abdominal distention, exostosis of the external auditory canal, feeding difficulties	In mice, acute knockdown of *Ipo8* leads to alteration of neuronal migration. The gene has been used as a house keeping gene in different tissues due to stable expression. Tissue expression includes heart, lung, adipose tissue, retina, and cancer cells	30042658, 29036603, 27339468, 29881787	–
**6**	*IPO8*	NM_006390.3:c.1933C>T	p.Gln645*	Hom	Yes, yes	Cutis laxa, joint hypermobility, generalized joint laxity, short stature, abnormal facial shape	As above	As above	–
**7**	*IPO8*	NM_006390.3: c.2695 + 4_2695 + 8del (NC_000012.11:g.30789908_30789912del)	p.?	Hom	Yes, no	Cutis laxa, joint hypermobility, umbilical hernia, high narrow palate, abnormal facial shape, mitral valve abnormality	As above	As above	–
**8**	*IPO8*	NM_006390.3:c.776G>A	p.Trp259*	Hom	Yes, no	Global developmental delay, hydronephrosis, muscular hypotonia	As above	As above	–
**9**	*IPO8*	NM_006390.3:c.776G>A	p.Trp259*	Hom	Yes, no	Brachycephaly, microcephaly, hypertelorism, low-set ears, blue sclerae, nystagmus, pectus excavatum, hypothyroidism, hirsutism, global developmental delay, joint laxity, failure to thrive, right ventricular hypertrophy, abnormal facial shape, pulmonary arterial hypertension, emphysema, pneumothorax, sparse scalp hair, tortuous cerebral arteries, right ventricular dilatation, depressed nasal bridge	As above	As above	–
**10**	*IPO8*	NM_006390.3: c.1881 + 1G>A (NC_000012.11:g.30814074C>T)	p.?	Hom	Yes, no	Hydronephrosis, macroglossia, deeply set eye, hyperextensible skin, muscular hypotonia, joint hypermobility, failure to thrive, ventricular septal defect, high, narrow palate, genu valgum, short stature, prominent superficial blood vessels, external ear malformation, bifid tongue	As above	As above	–
**11**	*IPO8*	NM_006390.3: c.2695 + 4_2695 + 8del (NC_000012.11:g.30789908_30789912del)	p.?	Hom	Yes, yes	Micrognathia, posteriorly rotated ears, macrotia, abnormal sclera morphology, muscular hypotonia, global developmental delay, hip dysplasia, joint laxity, polyhydramnios, atrial septal defect, gastroesophageal reflux, intestinal malrotation, abnormal branching pattern of the aortic arch, right aortic arch with retroesophageal left subclavian artery, epidural hemorrhage, malposition of the stomach	As above	As above	
**12**	*IPO8*	NM_006390.3:c.776G>A	p.Trp259*	Hom	Yes, yes	Congenital onset, failure to thrive, global developmental delay, mitral valve prolapse, muscular hypotonia, patent ductus arteriosus, patent foramen ovale, pulmonary arterial hypertension, ventricular septal defect, joint hypermobility	As above	As above	
**13**	*IPO8*	NM_006390.3:c.950A>G	p.Tyr317Cys	Hom	Yes, unknown	Abnormal facial shape, abnormal inflammatory response, atrial septal defect, bifid uvula, cleft soft palate, constipation, developmental regression, dysphagia, dyspnea, failure to thrive, feeding difficulties, fetal distress, gastroesophageal reflux, global developmental delay, intestinal malrotation, joint hypermobility, microretrognathia, muscular hypotonia	As above	As above	
**14**	*MMP15*	NM_002428.3:c.1058delC	p.Pro353fs	Hom	Yes, yes	Pointed chin, hypertelorism, wide nasal bridge, webbed neck, jaundice, cyanosis, cholestasis, ventricular septal defect, atrial septal defect, double outlet right ventricle, abnormal facial shape, elevated hepatic transaminases, congenital onset, hypoplastic left heart, tricuspid regurgitation	This gene may be involved in endothelial to mesenchymal transformation and endocardial cushion development via interaction with zinc-finger transcription factor Snai1	21920357	Cosegregation in affected sibling
**15**	*MMP15*	NM_002428.3:c.1058delC	p.Pro353fs	Hom	Sibling of above	Abnormal facial shape, antenatal onset, conjugated hyperbilirubinemia, decreased body weight, deeply set eye, failure to thrive, hypertelorism, intrauterine growth retardation, jaundice, left atrial enlargement, left ventricular hypertrophy, low-set ears, patent foramen ovale, pointed chin, prominent forehead, pulmonary arterial hypertension, tachycardia, triangular face, ventricular septal defect	As above	As above	As above
**16**	*MMP15*	NM_002428.3:c.691G>A	p.Gly231arg	Hom	Yes, no	Nephrocalcinosis, cholestasis, intrauterine growth retardation, ventricular septal defect, atrial septal defect, hepatomegaly, elevated hepatic transaminase	As above	As above	–
**17**	*PLK1*	NM_005030.5:c.785G>A	p.Arg262Gln	Hom	Yes, yes	Focal-onset seizure, seizures	The *WDR62* gene encodes a protein that localizes to the centrosome and to the nucleus, depending on the cell phase and on the cell type. WDR62 is a PLK1 substrate that is phosphorylated at Ser 897, and this phosphorylation at the spindle poles promotes astral microtubule assembly to stabilize spindle orientation. Primary microcephaly type 2 (MCPH2) is caused by homozygous or compound heterozygous variant in the *WDR62* gene	28973348	–
**18**	*PLK1*	NM_005030.5:c.785G>A	p.Arg262Gln	Hom	Yes, yes	Arachnoid cyst, cerebellar vermis hypoplasia, decreased body weight, delayed myelination, global developmental delay, jaundice, microcephaly, oligohydramnios, posterior fossa cyst, premature birth, seizures, short chin, short stature	As above	As above	–
**19**	*PLK1*	NM_005030.5:c.785G>A	p.Arg262Gln	Hom	Yes, yes	Generalized tonic–clonic seizures, global developmental delay, hyperpigmentation of the skin, microcephaly	As above	As above	–
**20**	*PLK1*	NM_005030.5:c.785G>A	p.Arg262Gln	Hom	Yes, no	Global developmental delay, microcephaly, intractable seizures	As above	As above	–
**21**	*PLK1*	NM_005030.5:c.408 + 3G>A (NC_000016.9:g.23690664G>A)	p.?	Hom	Yes, yes	Abnormal myelination, brain atrophy, epileptic encephalopathy, generalized-onset seizure, hyperreflexia, infantile encephalopathy, intellectual disability, leukodystrophy, leukoencephalopathy, microcephaly, muscular hypotonia, spasticity	As above	As above	–
**22**	*RAP1GDS1*	NM_001100426.1:c.1444-1G>A (NC_000004.11:g.99355086G>A)	p.?	Hom	Yes, no	Abnormality of midbrain morphology, mild microcephaly, motor delay, spastic paraplegia	The *RAP1GDS1* gene encodes for a protein that is involved in the stimulation of activities of small GTP-binding proteins (G proteins) including Rap1a/Rap1b, Rhoda, RhoB, and KRas	32727735, 32431071	–
**23**	*RAP1GDS1*	NM_001100426.1: c.83delT	p.Leu28fs	Hom	Yes, yes	Abnormal facial shape, abnormality of movement, decreased body weight, delayed myelination, developmental regression, difficulty standing, generalized hypotonia, global developmental delay, high forehead, highly arched eyebrow, hypertelorism, hypocystinemia, joint laxity, low-set ears, nasal flaring, otitis media, short stature, stereotypy	As above	As above	–
**24**	*RAP1GDS1*	NM_001100426.1:c.1444-1G>A (NC_000004.11:g.99355086G>A)	p.?	Hom	Yes, yes	Bulbous nose, central hypotonia, cryptorchidism, difficulty standing, difficulty walking, global developmental delay, heart murmur, inability to walk, jaundice, muscular hypotonia, retrognathia, smooth philtrum	As above	As above	This variant cosegregates in two unrelated families. Exon 13 skipping was detected in blood mRNA from the patients
**25**	*RAP1GDS1*	NM_001100426.1:c.1444-1G>A (NC_000004.11:g.99355086G>A)	p.?	Hom	Sibling of above	Motor delay, muscular hypotonia	As above	As above	As above
**26**	*ZNF699*	NM_198535.2: c.436_439del	p.Asp146Ilefs*10	Hom	Yes, yes	High palate, coarse facial features, smooth philtrum, long philtrum, abnormal eyelash morphology, proptosis, abnormal eyebrow morphology, hypotelorism, osteopenia, single umbilical artery, global developmental delay, agenesis of corpus callosum, cholestasis, vocal cord paralysis, premature birth, atrial septal defect, pulmonic stenosis, tachycardia, gastroesophageal reflux, hepatomegaly, recurrent infections, delayed skeletal maturation, decreased body weight, jejunal atresia, persistent left superior vena cava, bilateral sensorineural hearing impairment, protruding tongue, bilateral renal hypoplasia, abnormal spleen morphology, nasogastric tube feeding	This gene was found to be expressed ubiquitously in *Drosophila* nervous system, and the encoded protein localized in nuclei of the neurons	16094367	–
**27**	*ZNF699*	NM_198535.2: c.1623_1626delTTAT	p.Tyr542fs	Hom	Yes, no	Wide mouth, microcephaly, abnormality of the face, smooth philtrum, micrognathia, abnormal electroretinogram, long eyelashes, nystagmus, syndactyly, intellectual disability, hepatosplenomegaly, failure to thrive, intrauterine growth retardation, pancytopenia, anemia, malformation of the heart and great vessels, polydactyly, abnormal renal cortex morphology, abnormal renal medulla morphology	As above	As above	–
**28**	*ZNF699*	NM_198535.2: c.1623_1626delTTAT	p.Tyr542fs	Hom	Yes, no	Cryptorchidism, retrognathia, triangular face, macrotia, prominent nasal bridge, syndactyly, intellectual disability, global developmental delay, abnormal facial shape	As above	As above	–
**29**	*ZNF699*	NM_198535.2: c.1324dupA	p.Ser442fs	Hom	Yes, yes	Microcephaly, intellectual disability, global developmental delay, hiatus hernia, developmental regression, recurrent infections, immunodeficiency, tracheomalacia, bronchomalacia, chronic lung disease, short thumb, intestinal atresia, feeding difficulties, bilateral renal dysplasia, chronic kidney disease	As above	As above	–
**30**	*ZNF699*	NM_198535.2: c.349dupA	p.Ile117fs	Hom	Yes, yes	Syndactyly, muscular hypotonia, intrauterine growth retardation, premature birth, abnormal facial shape, congenital onset, intestinal atresia	As above	As above	–
**31**	*ZNF699*	NM_198535.2: c.436_439del	p.Asp146Ilefs*10	Hom	Yes, yes	2–3 toe syndactyly, abnormal cry, abnormal eyebrow morphology, antenatal onset, anteverted nares, chronic kidney disease, failure to thrive, generalized hypotonia, hypertrichosis, intrauterine growth retardation, jejunal atresia, long eyelashes, long philtrum, low anterior hairline, low posterior hairline, microcephaly, preaxial hand polydactyly, renal hypoplasia, short nose, synophrys, ventricular septal defect	As above	As above	–
**32**	*ZNF699*	NM_198535.2: c.436_439del	p.Asp146Ilefs*10	Hom	Yes, no	Abnormal facial shape, diaphragmatic eventration, failure to thrive, fever, generalized hypotonia, global developmental delay, hemihypertrophy, intestinal atresia, iron deficiency anemia, microcephaly, oral–pharyngeal dysphagia, pallor, ptosis, sensorineural hearing impairment	As above	As above	–
**33**	*ZNF699*	NM_198535.2: c.436_439del	p.Asp146Ilefs*10	Hom	Yes, yes	Abnormal eyelash morphology (partially hypopigmented), abnormality of skin pigmentation, hypopigmentation of hair, intellectual disability, failure to thrive, anemia, abnormal facial shape, congenital hypoplastic anemia	As above	As above	–
**34**	*ZNF699*	NM_198535.2: c.436_439del	p.Asp146Ilefs*10	Hom	Yes, yes, sibling above	Similarly affected as index	As above	As above	–
**35**	*ZNF699*	NM_198535.2: c.436_439del	p.Asp146Ilefs*10	Hom	Yes, no	Abnormal facial shape, abnormal myelination, absent thumb, ambiguous genitalia, anteverted nares, cryptorchidism, dysplastic pulmonary valve, hyperbilirubinemia, hypertelorism, hypospadias, intestinal atresia, muscular hypotonia, polyhydramnios, posteriorly rotated ears, premature birth, pulmonary arterial hypertension, pulmonic stenosis, reduced blood folate concentration, sacral dimple, short nose, small for gestational age, thick vermilion border, ventriculomegaly, wide intermammary distance	As above	As above	–
**36**	*ZNF699*	NM_198535.2: c.51_54delCTCA	p.Asp17fs	Hom	Yes, yes	Cryptorchidism, chordee, ambiguous genitalia, abnormality of the face, micrognathia, low-set ears, syndactyly, craniosynostosis, patent foramen ovale, abnormal facial shape, jejunal atresia, echogenic fetal bowel	As above	As above	–
**37**	*ZNF699*	NM_198535.2: c.436_439del	p.Asp146Ilefs*10	Hom	Yes, no	Abnormal facial shape, microcephaly, hearing impairment, macrotia, prominent nasal bridge, prominent nose, microphthalmia, abnormal thumb morphology, intellectual disability, muscular hypotonia, talipes equinovarus, pancytopenia, fever, pneumonia, asthma, premature graying of hair, immunodeficiency, genu valgum, 2–3 toe syndactyly, feeding difficulties, rhinitis, choking episodes, hemihypotrophy of lower limb	As above	As above	–
**38**	*ZNF699*	NM_198535.2:c.436_439del	p.Asp146Ilefs*10	Hom	Yes, yes	2–3 toe syndactyly, abnormal facial shape, anemia, breech presentation, caesarean section, coarse facial features, failure to thrive, feeding difficulties, global developmental delay, jejunal atresia, laryngomalacia, leukopenia, microcephaly, muscular hypotonia, nasogastric tube feeding, plagiocephaly, poor suck, premature birth, pyloric stenosis, recurrent infections, recurrent urinary tract infections, tracheomalacia, unilateral conductive hearing impairment, atrial septal defect and patent ductus arteriosus	As above	As above	–

Patients’ phenotype (Human Phenotype Ontology [HPO]) and related published evidence are summarized. NC_XXX nomenclature is used for intronic variants according to Human Genome Variation Society (HGVS) guidelines.

*cDNA* complementary DNA, *CHD* congenital heart defect, *CM* cardiomyopathy, *Hom* homozygote, *mRNA* messenger RNA.

The related disorders are described as follows: three different neurodevelopmental disorders with features of (1) severe ID, leukodystrophy, seizures, and visual impairment (*BLOC1S1*, four patients); (2) NDD, seizures, and microcephaly (*PLK1*, five patients); and (3) NDD, dysmorphic features, and hypotonia (*RAP1GDS1*, four patients). We also identified three new syndromic associations: (1) a connective tissue disorder resembling Loeys–Dietz syndrome (LDS) (*IPO8*, nine patients); (2) Alagille-like syndrome with liver cholestasis and congenital heart defects (*MMP15*, three patients); and (3) a multiple malformation syndrome (*ZNF699*, 13 patients). Variant details and patient phenotypes are summarized in Table 1. All cases presented homozygous variants compatible with autosomal recessive disorders. Selected examples are described below.

### *BLOC1S1*

Four patients from three families presented rare homozygous variants in this gene and a similar neurological phenotype. Additional testing in one family confirmed cosegregation of the variant in two siblings (affected sibling—homozygote and unaffected sibling—heterozygote). BLOC1S1 is a component of the ubiquitously expressed BLOC1 multisubunit protein complex, which is required for normal biogenesis of specialized organelles of the endosomal–lysosomal system.^22^ The gene was originally identified as *GCN5L1*; it has been shown to play crucial roles in mitochondria, endosomes, lysosomes, and synaptic vesicle precursors.^23^ Knocking out this gene in mice results in lethality; mice embryos fail to develop beyond ∼E12.5.^24^ Furthermore, mutant flies lacking the conserved Blos1 subunit displayed eye pigmentation defects, as well as abnormal glutamatergic transmission and behavior.^25^ BLOC1S3 is another component of the ubiquitously expressed BLOC1 multisubunit protein complex. Biallelic pathogenic variants in *BLOC1S3* cause Hermansky–Pudlak syndrome (OMIM 614077), a sort of incomplete oculocutaneous albinism and platelet dysfunction that includes visual defects.

### *IPO8*

**Fig. 2:**
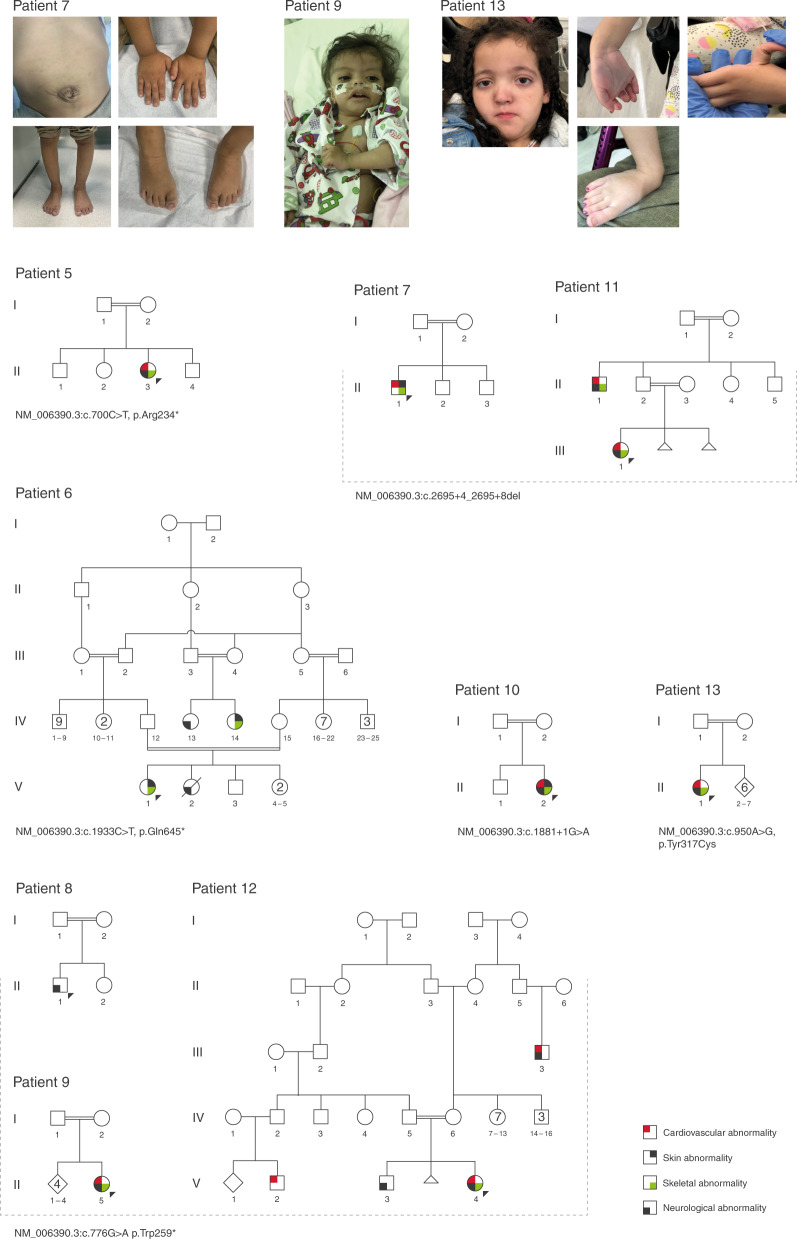
The phenotype associated with *IPO8* homozygous loss-of-function (LoF) variants. Upper panel: photographs illustrating clinical features. Patient 7, with umbilical hernia, brachydactyly of hands, proximal placement of the thumbs, short nails, genus varus, pes planus, brachydactyly of foot, and short toenails. Patient 9 with low-set ears, sparse scalp hair, broad and sparse eyebrows, hypertelorism, long palpebral fissures, and depressed nasal bridge. Patient 13 with frontal bossing, wide, sparse eyebrows, hypertelorism, large palpebral fissures (downslanted), deep philtrum, and thin vermilion of the upper lip. Joint hypermobility (wrist and thumb), as well as long foot, long toes, hindfoot valgus, and pes planus. Lower panel: family trees of patients with *IPO8* homozygous LoF variants and clinical abnormalities. Patients presented with a complex phenotype that included abnormalities of the cardiovascular system (congenital heart defects, cardiomyopathy, engorged brain vasculature), the skeletal system (joint hypermobility, pectus deformities, genus valgus/varus, scoliosis), and the skin (cutis laxa). Most patients presented hypotonia, neurodevelopmental delay (NDD), and failure to thrive. Other features included intestinal malrotation, Gastroesophageal reflux (GER), and hydronephrosis.

Six different homozygous LoF variants were identified in the *IPO8* gene in nine unrelated patients (Table 1 and Fig. 2). Phenotypically, the patients presented dysmorphic features, hypotonia, and features reminiscent of a connective tissue disease such as high palate, pectus deformities, hernias, gray-blue sclera, cutis laxa, tortuosity of cerebral arteries, and congenital heart defects. For some cases, clinical suspicion included LDS and Ehlers–Danlos syndrome. The *IPO8* gene has not been associated to any human phenotype so far. Interestingly, Imp8 is involved in preferential nuclear importing of Smad1, Smad3, and Smad4. The TGFB pathway and receptor SMADs (SMAD2/3) are central in the pathophysiology of LDS with causative variants detected in the *TGFBR2/3*,^26^ *TGFB2/3*,^27,28^ *SMAD2/3*.^29,30^

### *MMP15*

Upon the detection of the homozygous variant NM_002428.3:c.1058delC, p.Pro353fs in a patient with dysmorphic features, complex congenital heart defects (double outlet of the right ventricle, hypoplastic left ventricle, septal defects), and cholestasis, we queried our data repository for additional cases. A sibling was similarly affected and was homozygote for the same variant. An additional unrelated patient was identified with a different variant in the *MMP15* gene (Table 1). The patient presented cholestasis, hepatomegaly, high hepatic transaminases, and congenital heart disease. Alagille syndrome and progressive familial intrahepatic cholestasis were the differential diagnoses. MMP15, a member of the matrix metalloproteinases family, is an excellent candidate for this phenotype. In mice, Mmp15 is a direct target of Snail1 during endothelial to mesenchymal transformation and endocardial cushion development.^31^ A Snail1/Notch1 signaling axis controls embryonic vascular development. Snail1 acts as a VEGF-induced regulator of Notch1 signaling and Dll4 expression.^32^ In humans, genes from the *NOTCH* pathway (*JAG1* and *NOTCH2*) are implicated in Alagille syndrome type 1 and 2 (OMIM 118450 and 610205), which has high similarity with the phenotype described here in patients with homozygous variants in *MMP15*. Interestingly, while these syndromes present with an autosomal dominant mode of inheritance, the patients reported in this study with *MMP15* variants show an autosomal recessive disease.

### *ZNF699*

**Fig. 3:**
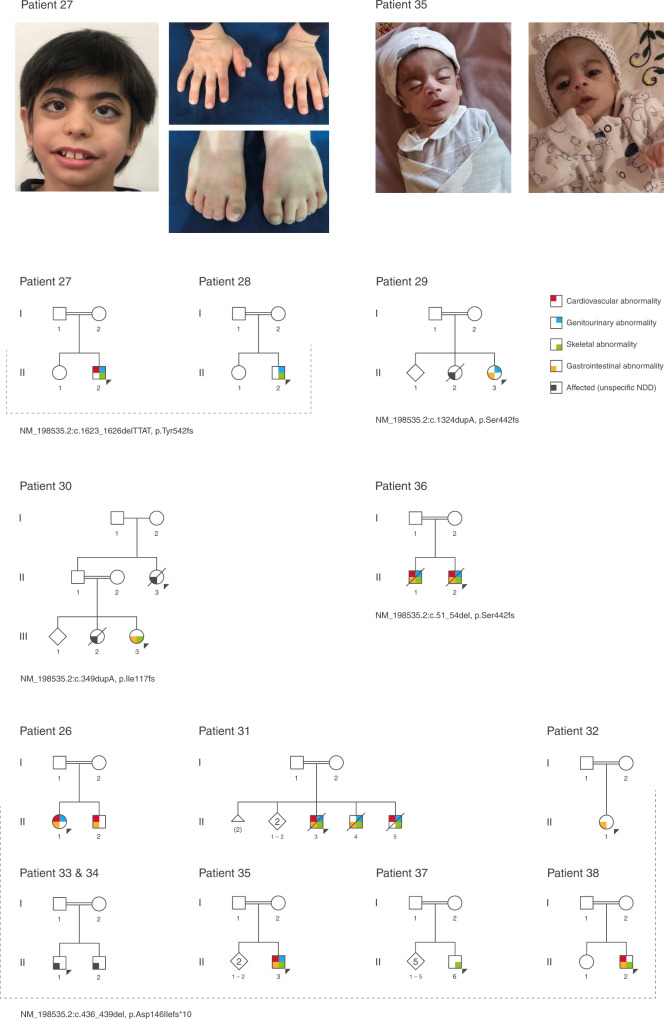
The phenotype associated with *ZNF699* homozygous loss-of-function (LoF) variants. Upper panel: photographs showing phenotypic features of two patients (patients 27 and 35, Table 1). Patient 27, male index has low anterior hairline, thick scalp hair, thick eyebrows, synophrys, long eyelashes, long palpebral fissures, proptosis, strabismus, bulbous nose, low hanging columella, smooth philtrum, wide mouth, micrognathia, short neck, brachydactyly, right preaxial polydactyly, and bilateral syndactyly of the second and third toes. Patient 35, male index with coarse face, broad eyebrows, long palpebral fissures, wide mouth, thin vermilion of the upper lip, and bilateral absent thumbs. He presented generalized hypotonia and was severely emaciated. The patient deceased at 9 months old. Lower panel: summarized family trees of patients with *ZNF699* homozygous LoF variants and clinical features. Patients presented with a severe phenotype that included congenital heart defects, gastrointestinal (intestinal atresia, pyloric stenosis, GER, hepatosplenomegaly), genitourinary (renal hypoplasia, cryptorchidism, chordee, hypospadias, ambiguous genitalia), and skeletal abnormalities (preaxial polydactyly, absent thumbs, syndactyly). Other recurrent features were generalized hypotonia, sensorineural hearing impairment, and premature hair graying. All patients have severe neurodevelopmental delay (NDD).

Thirteen patients from 12 families were identified with homozygous loss-of-function (LoF) variants in this gene (Fig. 3). These patients presented with a clear malformation syndrome with coarse facial features and abnormalities of the cardiovascular, gastrointestinal (gastroesophageal reflux, intestinal atresia), genitourinary (renal dysplasia/hypoplasia, ambiguous genitalia), and skeletal system (syndactyly, preaxial polydactyly, absent thumbs). Other common features included anemia/pancytopenia, premature graying of hair, and sensorineural hearing impairment. All patients presented severe NDD.

The first patient identified was a 2-year-old female born preterm (32 weeks) to consanguineous parents (patient 26, Table 1, Fig. 3). She has a similarly affected sibling, who is also homozygote for the same *ZNF699* variant. A clinical summary of the patients from three families is presented in the Supplementary Information (patients 26, 35, and 38, Table 1, Fig. 3). Despite the clear phenotypical similarity of the 13 patients identified with homozygous LoF variants in *ZNF699*, little is known about the function of this gene, which was initially described in *Drosophila* in a study of alcohol dependence.^33^ The gene encodes a large nuclear zinc-finger protein, suggesting a molecular role in nucleic acid binding.^34^

We also detected variants in known candidate genes that had insufficient published evidence supporting causality (and no OMIM associated phenotypes). Our current data provides further evidence supporting confirmation of 31 candidate genes in 56 patients with a wide range of clinical phenotypes. These include cases with syndromic and nonsyndromic forms of NDD/ID, ciliopathy, oral–facial–digital syndrome, cardiomyopathy, syndromic short stature, and skeletal dysplasia. The identified genes are *APC2*, *CAP2*, *EIF3F*, *GYG2*, *IFT57*, *ITFG2*, *LGI3*, *NEK10*, *NRAP*, *PAPPA2*, *PPP1R13L*, *WIPI2*, *ZNF526* (autosomal recessive, X-linked inheritance, Supplementary table 1); *AFF3*, *BCORL1*, *CHD6*, *CNOT1*, *CTR9*, *DMXL1*, *FRYL*, *KLF7*, *MYCBP2*, *NRXN2*, *PHF21a*, *RAB11a*, *RALA*, *SPEN*, *TAF4*, *TANC2*, *ZNF292*, *ZNF462* (autosomal dominant, de novo variants, Supplementary Table 2). Selected examples from this group are described in the following sections.

### *PAPPA2*

Dauber et al. reported the finding of two homozygous variants (missense and frameshift) in two unrelated families, with several children having significant postnatal growth retardation, long thin bones, long fingers and toes, mild microcephaly, abnormal dentine and teeth enamel, and mild dysmorphisms. In vitro analyses demonstrated that both variants caused a complete absence of PAPPA2 proteolytic activity;^35^ however, no additional patients have been reported to date. We identified two novel homozygous nonsense variants in *PAPPA2*, in two patients with short stature and dysmorphic features with no evident NDD. The phenotype is highly similar to the previously reported cases supporting a causal role of *PAPPA2* in a novel short stature syndrome.

### *TAF4*

A heterozygous de novo variant (frameshift) was reported in *TAF4* by Kosmicki et al., in a patient with autism.^36^ The gene has no phenotypic association in OMIM (accessed 12 October 2020). Within this study, we identified two additional de novo LoF variants (splicing and nonsense) in two unrelated patients with dysmorphic features and NDD. *TAF4* is highly intolerant to LoF as documented in gnomAD (pLi = 1). Expression of *TAF4* varies during development and in the processes of cell differentiation; *TAF4* is detected in various regions of the human brain, and it is believed to control the differentiation of human neural progenitor cells having a role in the regulation of neural development and brain function.^37^ The current data suggests that *TAF4* haploinsufficiency leads to NDD in humans.

### *RAB11a*

Hamdan et al. described three patients with developmental and epileptic encephalopathy as well as de novo missense variants in the *RAB11a* gene.^38^ We identified two additional variants in the same GTPase region of *RAB11a* in patients with microcephaly, NDD, and specific brain abnormalities. Dendritic spines are postsynaptic protrusions at excitatory synapses that are critical for proper neuronal synaptic transmission. *RAB11a* is part of the cascade controlling spine formation and function.^39^ When combined, the genetic and functional data support a causative role of *RAB11a* for NDD with epileptic encephalopathy and microcephaly.

### *MYCBP2*

This gene is not associated to any phenotype in OMIM (accessed 12 October 2020). Neale and Kosmicki et al.^36,40^ reported de novo missense and frameshift variants in patients with autism spectrum disorder after screening a large cohort of patients. Recently, Takahashi et al. identified two variants (one of them confirmed as arisen de novo) in two cases with uterovaginal aplasia with concomitant defects, such as renal, skeletal malformations, hearing defects, and rare cardiac and digital anomalies known as Mayer–Rokitansky–Küster–Hauser (MRKH) syndrome.^41^ Within this study, we detected three additional de novo variants (one likely affecting splicing and two missense) in three patients with NDD, microcephaly, and seizures. One case presented bilateral bifid thumbs, talipes, and scoliosis, without vaginal or uterine anomalies (two female patients, both were prepubertal). Our results support a causal link of *MYCBP2* de novo variants and ID/NDD.

## DISCUSSION

The ACMG/AMP guidelines for the interpretation of genetic variants are restricted to genes with established causality in human diseases,^12^ while variants in genes for which this evidence is insufficient are considered genes of unknown significance (“research” or “candidate” genes).^12^ Therefore, in routine diagnostics, many genes are excluded during the filtering process of exome/genome data.

Clear guidelines should be established to identify, classify, and report variants located in candidate genes. Recently, Strande et al.^42^ proposed a comprehensive framework within the ClinGen initiative to evaluate relevant genetic and functional evidence supporting or contradicting gene–disease associations. The curation system covers gene variant evidence based on genetic data, and functional or experimental evidence. Gene-supporting evidence includes the identification of several unrelated patients and variants, and the absence of contradicting data (i.e., high variant frequency in controls).^42^ Experimental evidence comprises data on gene function, and cellular and animal models.^42^

As part of this study, we describe a patient-centered workflow implemented for cases with inconclusive or no genetic diagnosis after ES/GS. The process extends the search and evaluation to variants detected in genes of unknown significance. From these, we suggest six novel disease–gene associations. The findings are exclusively based on the analyses performed on our data repository, which enabled further identification of unrelated patients displaying similar phenotypes. As a follow-up, functional work is needed to confirm and to understand the disease mechanisms and related pathophysiology. This is particularly relevant for genes such as *IPO8* and *ZNF699*, as little is known about their function. For both genes, the high number of affected individuals identified, the similarities of their phenotype, and the putative LoF nature of the homozygous variants detected are compelling evidence favoring a gene–disease association. Furthermore, our results support causality of 31 additional candidate genes. Following the ClinGen guidelines, these 31 gene–disease associations can be upgraded from having “limited” evidence to genes with “moderate” or “strong” evidence, based on 56 patients.

Traditionally, discovery of novel gene–disease associations has been done by research labs; however, with this work, we show the enormous potential of diagnostic labs to uncover and validate candidate genes. Multiple strategies can be implemented to help identify novel disease genes, which will ultimately benefit the patients and families with rare genetic diseases. Genomic data analysis beyond known disease genes can be implemented in a routine diagnostic approach, as shown within this study. Finally, for genetic labs, reporting of variants in diagnostic genes versus candidate genes should be clearly differentiated since clinical validity is restricted to the former. Communication with referring physicians are critical for follow-up and further validation of the gene–disease associations.

In conclusion, our work shows the benefits of performing extended ES/GS analyses in patients with no genetic diagnosis combined with further data repository mining. Dedicated analyses of such data repositories that combine clinical and genetic information can be routinely performed to identify and confirm candidate genes. Genetic laboratories should be encouraged to pursue such analyses for the benefit of undiagnosed patients and their families.

## Supplementary information

Supplementary file

Supplementary Table1_2

## Data Availability

The data set that was generated and/or analyzed as part of this study is available from the corresponding author.
